# Hsa-miR-105-1 Regulates Cisplatin-Resistance in Ovarian Carcinoma Cells by Targeting ANXA9

**DOI:** 10.1155/2021/6662486

**Published:** 2021-02-24

**Authors:** Xinxin Kou, Hui Ding, Lei Li, Hongtu Chao

**Affiliations:** Department of Gynaecology, Cancer Hospital Affiliated to Zhengzhou University, Zhengzhou, China

## Abstract

**Purpose:**

Cisplatin is one of the most effective drugs for treating ovarian carcinoma (OC), which is among the most lethal types of carcinoma. However, the chemoresistance to cisplatin that develops over time leads to a poor clinical outcome for many OC patients. Therefore, it is necessary to clearly understand the molecular mechanisms of chemoresistance. In this study, we examined how Hsa-miR-105-1 functions in cisplatin-resistant OC cells.

**Methods:**

The levels of Hsa-miR-105-1 expression in cisplatin-sensitive and resistant OC cell lines were detected by qRT-PCR. The target gene of Hsa-miR-105-1 was predicted by using the TargetScan and Starbase databases and verified by the double luciferase reporter gene assay. The target gene of Hsa-miR-105-1 was identified as *ANXA9*, and *ANXA9* expression was evaluated by qRT-PCR, western blotting, and immunofluorescence. To validate the function of Hsa-miR-105-1 in OC cells, we silenced or overexpressed Hsa-miR-105-1 in cisplatin-sensitive or resistant OC cell lines, respectively. Furthermore, the expression levels of several apoptosis-related proteins, including P53, P21, E2F1, Bcl-2, Bax, and caspase-3, were examined by western blot analysis.

**Results:**

The levels of Hsa-miR-105-1 expression were abnormally downregulated in cisplatin-resistant OC cells, while *ANXA9* expression was significantly upregulated in those cells. Treatment with an Hsa-miR-105-1 inhibitor promoted the expression of ANXA9 mRNA and protein, enhanced the resistance to cisplatin, and attenuated the cell apoptosis induced by cisplatin in cisplatin-sensitive OC cells. Moreover, treatment with Hsa-miR-105-1 mimics inhibited *ANXA9* expression, which further increased the levels of P53, P21, and Bax expression and decreased the levels of E2F1 and Bcl-2 expression, finally resulting in an increased sensitivity to cisplatin in cisplatin-resistant OC cells.

**Conclusion:**

We found that a downregulation of Hsa-miR-105-1 expression enhanced cisplatin-resistance, while an upregulation of Hsa-miR-105-1 restored the sensitivity of OC cells to cisplatin. The Hsa-miR-105-1/*ANXA9* axis plays an important role in the cisplatin-resistance of OC cells.

## 1. Introduction

Ovarian carcinoma (OC), which can be divided into subtypes of epithelial ovarian carcinoma, germinoma, ovarian sex gonad stromal tumors, and metastatic tumors based on tissue origin, is one of the most lethal gynecologic tumors worldwide [1]. More than 220 thousand women are diagnosed with OC and 140 thousand die from it annually. Its treatment usually consists of optimal cytoreductive surgery and platinum-based combination chemotherapies. Cisplatin, also referred to as the “penicillin of cancer,” has played a critical role in treating cancers since 1971. Cisplatin is an alkylating agent that causes the formation of inter- and intrastrand DNA cross-links, which finally leads to cell cycle arrest. While cisplatin is among the most effective anticancer drugs used in the treatment of ovarian cancer, innate and acquired resistance to cisplatin is frequently observed. As a result, the 5-year survival rate of advanced ovarian cancer patients (stages III and IV) is only 20–30%. It has become increasingly apparent that the early detection of drug resistance and prompt adjustment of a patient's chemotherapy regimen are critically important for improving the clinical outcomes of patients with ovarian cancer.

MicroRNAs (miRNAs) comprise a group of evolutionarily conserved small noncoding RNAs that regulate gene expression at the transcriptional or posttranscriptional level [2]. MicroRNAs play important roles in many biological processes, such as cell proliferation, apoptosis, migration, invasion, differentiation, and metabolism [3]. Numerous studies have demonstrated that miRNAs are involved in the chemoresistance developed by various cancers [4, 5]. A search of the GSE dataset revealed that miR-105-1 is expressed at significantly higher levels in noncisplatin-resistance cancer cells when compared to cisplatin-resistance cancer cells (data not shown). Therefore, we speculated that miR-105-1 might play an important role in the chemoresistance to cisplatin developed by ovarian carcinoma tumors. Annexin A9 (ANXA9), a member of the annexin protein family, has been reported to be involved in the invasion and metastasis of various cancer cells, such as colorectal cancer (CRC) [6], breast cancer [7, 8], and head and neck squamous cell carcinoma cells [9]. However, few studies have investigated the role of ANXA9 in OC. In this study, we examined the roles played by Hsa-miR-105-1 and ANXA9 in the development of cisplatin-resistance in OC cells.

## 2. Materials and Methods

### 2.1. Cell Culture

Five OC cell lines (SW626, SK-OV-3, TOV-112D, A2780, and OVcar3) and a normal ovarian epithelial cell line (IOSE-80) were purchased from Nanjing KeyGen Biotech (Nanjing, China). All the cell lines were initially stored in liquid nitrogen and subsequently allowed to recover in a complete DMEM medium (Thermo Fisher Scientific, Waltham, MA, USA) containing 10% fetal bovine serum, 10,000 UI/mL penicillin, and 10 mg/mL streptomycin prior to use. The cells then were cultured in a humidified 37°C incubator containing 5% carbon dioxide.

### 2.2. Establishment of an Acquired Cisplatin-Resistant OC Cell Model

To establish stable cisplatin-resistant OC models, SW626 and OVcar3 cells were exposed to stepwise increasing concentrations of cisplatin (Sigma-Aldrich, Darmstadt, Germany). Briefly, 1 × 10^6^ SW626 or OVcar3 cells were cultured in 6 mm plates and initially treated with 0.1 *μ*M cisplatin (a concentration below their respective IC_50_) for 1 month. Subsequently, the cisplatin concentration in the culture medium was increased by 0.1 *μ*M every 2 weeks up to a final concentration of 1 *μ*M (until a cell population was selected that demonstrated at least a 3-fold higher IC_50_ (45 *μ*g/mL) to cisplatin than the parental cell lines). In parallel, control wild type cells were treated in the same manner but without cisplatin. The established cisplatin-resistant SW626 and OVcar3 cells were designated as SW626/DDP and OVcar3/DDP, respectively. To eliminate the influence of residual cisplatin in culture medium, SW626/DDP and OVcar3/DDP cells were cultured in cisplatin-free DMEM supplementary medium for 2 weeks prior to their use in experiments.

### 2.3. Transfection

An Hsa-miR-105-1 inhibitor and Hsa-miR-105-1 mimics were synthesized by GenePharma Company (Shanghai, China). For transfection, cells were seeded into culture plates and transfected by using Lipofectamine TM2000 (Invitrogen, Carlsbad, CA, USA) according to the manufacturer's instructions.

### 2.4. Prediction of the Hsa-miR-105-1 Target Genes

The target genes for Has-miR-105-1 were predicted using the TargetScan and Starbase databases. Genes that were simultaneously predicted by both databases were identified as target genes for Hsa-miR-105-1. The potential binding site was then predicted.

### 2.5. Double Luciferase Reporter Gene Assay

Wild-type *ANXA9* fragments containing potential binding sites for Hsa-miR-105-1 were amplified from human genomic DNA by using PrimeSTAR® HS.DNA Polymerase (Takara, Japan). The primer sequences were as follows: 5′-CCCTCGAGTGAAACTGAGCCCAATTACCAAG-3′ and 5′-ATTTGCGGCCGCAGTGAGGCAGAAACTATCAAAGA-3′. Seed region mutagenesis was achieved by using the following mutant reverse primers: 5′-TCCCTGCCCCACCCCACATGTGTAGGCTGGATCTGAGATTTCCGTGTT-3′ and 5′-AACACGGAAATCTCAGATCCAGCCTACACATGTGGGGTGGGGCAGGGA-3′. Fragments containing the wild-type 3′UTR of *ANXA9* (*ANXA9*-3′UTR-WT) and mutant 3′UTR of *ANXA9* (*ANXA9*-3′UTR-MUT) were inserted into a downstream region of the psiCHECK-2 promoter vector luciferase gene, yielding the recombinant plasmids psiCHECK-2-*ANXA9*-3′UTR-WT and psiCHECK-2-*ANXA9*-3′UTR-MUT, respectively. For the luciferase reporter assay, the psiCHECK-2-*ANXA9*-3′UTR-WT or psiCHECK-2*-ANXA9*-3′UTR-MUT, Hsa-miR-105-1 mimics or mimic NC (negative control), and reporter plasmid pRL-TK with Renilla luciferase activity were transiently cotransfected into cells using Lipofectamine™ 2000 (Life Technologies, Carlsbad, CA, USA). After 24 hours of transfection, a dual-luciferase reporter assay system (Promega, Madison, WI, USA) was used to detect luciferase activity according to the manufacturer's protocol.

### 2.6. Quantitative Real-Time PCR (qRT-PCR)

Total RNA was extracted by using an RNeasy Mini Kit (Qiagen, Germany) according to the manufacturer's instructions and then reverse transcribed into cDNA using a Prime Script™ RT reagent Kit with gDNA Eraser (Takara, Japan). Small RNA was reverse transcribed into cDNA using a Mir-X miRNA First-Strand Synthesis Kit (Takara, Japan). The conditions used for qRT-PCR were as follows: 95°C for 5 min, 40 cycles of 95°C for 15 s and 60°C for 50 s, 95°C for 15 s, 60°C for 15 s, and 95°C for 15 s. The levels of Hsa-miR-105-1 and *ANXA9* were detected using SYBR Advantage qPCR Premix (Takara, Japan) on a 7900 HT Fast Real-Time PCR System (Applied Biosystems, Foster City, USA). RNU6-1 (U6) and GAPDH were used as internal controls for estimating Hsa-miR-105-1 and *ANXA9* levels, respectively. The primer pairs used for qRT-PCR were as follows: Hsa-miR-105-1, (F) 5′-ACACTCCAGCTGGGACGGATGTTTGAGCATGT-3′ and (R) 5′-CTCAACTGGTGTCGTGGA-3′; U6, (F) 5′-CTCGCTTCGGCAGCACA-3′ and (R) 5′-AACGCTTCACGAATTTGCGT-3′; *ANXA9*: (F) 5′-CAGCTCATCTCACGAAACTTCC-3′ and (R) 5′-GGTTCGAGTGGCAAGAATTTCAA-3′; GADP, (F) 5′-TGTTCGTCATGGGTGTGAAC-3′ and (R) 5′-ATGGCATGGACTGTGGTCAT-3′. Each PCR analysis was performed in triplicate. Relative levels of gene expression were calculated using the 2^−*ΔΔ*Ct^ method.

### 2.7. Western Blotting

Cells were lysed with 150 *μ*L of lysis buffer (Beyotime, China) containing 1% protease inhibitors (Thermo Fisher Scientific) on ice for 5 min and then washed twice with ice-cold PBS. Next, the cells were harvested and centrifuged at 12,000 g for 5 min at 4°C, and the protein concentration in each supernatant was determined using a BCA Kit (Beyotime). An equal amount of total protein (20 *μ*g) from each supernatant dissolved in 20 *μ*L of loading buffer (Beyotime) and separated by sodium dodecyl sulfate–polyacrylamide gel electrophoresis (Beyotime). The separated protein bands were transferred onto polyvinylidene difluoride (Roche Applied Science, Germany) membranes, which were subsequently blocked with 5% nonfat dry milk for 1 h at room temperature. Immunoblotting was carried out by incubating the membranes with primary antibodies against ANXA9, E2F1, Bcl-2, and Bax (1 : 500 dilution each), P53 and P21 (1 : 1000 dilution each), and caspase-3 and GAPDH (1 : 2000 dilution each) at room temperature for 1 h. After incubation with the primary antibodies, the membranes were washed and then incubated with an HRP-linked secondary antibody (1 : 5,000 dilution) at room temperature for 1 h. The immunostained protein bands were detected with enhanced chemiluminescence reagent (Biosharp, China), and images were captured with a chemiluminescence camera (Tanon, China). The staining intensity of each band was measured using ImageJ software (National Institutes of Health, USA). GAPDH from the same sample served as an internal loading control. Three independent experiments were performed.

### 2.8. Immunofluorescence

Treated cells were fixed with a mixture of 4% paraformaldehyde (PFA) and 0.0075% glutaraldehyde for 15 min; after which, they were permeabilized with phosphate buffer solution containing 0.1% Triton X-100 (PBST) and blocked with 10% BSA (Sigma-Aldrich, St. Louis, MO, USA) for 30 min at room temperature. After an overnight incubation at 4°C with antimouse ANXA9 (diluted 1 : 500 in PBST), the cells were washed with PBST (3× for 5 min) and then incubated with Alexa FluorR 488 conjugate [F(ab′)2-Goat antimouse IgG (H + L) cross-adsorbed secondary antibody diluted in PBST for 1 h at room temperature. The cell nuclei were then counterstained with 4′, 6-diamidino-2-phenylindole (DAPI), and the cells were mounted onto glass slides. Images were obtained with a fluorescence microscope (Olympus, Japan).

### 2.9. Cell Viability

Treated cells suspended in culture medium were seeded into 96-well plates at a density of 5000 cells/well and cultured for 24 h. The culture medium was then replaced with fresh medium containing 10% CCK-8 solution (Beyotime). After incubation for 1 h, the viability of the OC cells was detected by measuring the absorbance of each well at 450 nm with a fluorescence microplate reader (Tecan Infinite 200 PRO, Switzerland). The IC_50_ value was defined as the drug concentration that reduced the number of viable cells by 50% when compared with control cells and was calculated using GraphPad Prism 6.0 software (GraphPad Software, USA). Three culture wells were used for each treatment group, and three independent experiments were performed.

### 2.10. Flow Cytometry

For cell cycle assays, cells (~1 × 10^6^ cells per well) were harvested following different treatments and then fixed overnight in 70% ethanol at 4°C. Following fixation, the cells were centrifuged at 1,000 g for 5 min to remove the ethanol; after which, they were washed and subsequently stained with propidium iodide (PI) (10 *μ*g/mL). The cells were then treated with RNase A (100 *μ*g/mL) at room temperature for 30 min. For annexin V-fluorescein isothiocyanate (FITC)/PI double-staining assays, cells (~1 × 10^6^ cells per well) were collected and centrifuged at 1,000 g for 5 min at room temperature; after which, they were resuspended in ice-cold PBS, centrifuged at 1,000 g for 5 min, and washed. Next, the washed cells were resuspended in 500 *μ*L of 1 × binding buffer, and 5 *μ*L of annexin V–fluorescein isothiocyanate (FITC) staining solution plus 5 *μ*L of PI staining solution was added to the suspension. The suspended cells were then thoroughly mixed and incubated for 30 min at room temperature. The treated cells were analyzed with a BD FACS Calibur flow cytometer (Becton–Dickinson, Franklin Lakes, NJ, USA), and the distribution of cells in different phases of the cell cycle and the cell apoptosis rate were determined using FlowJo software (FlowJo LLC, Ashland, OR, USA). Three independent experiments were performed.

### 2.11. Hoechst Staining

Cells were seeded into 12-well plates at a density of 1 × 10^5^ per well. After being treated, the cells were washed twice with PBS, fixed with 4% PFA for 20 min, and then washed twice again with PBS. The fixed cells were then incubated with Hoechst 33342 staining solution (Invitrogen, USA) for 5 min, and cellular fluorescence was observed under a microscope (Olympus, Japan).

### 2.12. Statistical Analysis

All data were analyzed using IBM SPSS Statistics for Windows, Version 19.0 software (IBM Corp, Armonk, NY USA), and results are expressed as a mean value ± SD (*n* = 3). Comparisons between two groups were performed using Student's *t*-test, and comparisons among multiple groups were performed using one-way analysis of variance followed by the Student–Newman–Keuls *post hoc* test. A *P* value < 0.05 was considered to be statistically significant.

## 3. Results

### 3.1. Hsa-miR-105-1 Expression Was Downregulated in Cisplatin-Resistant OC Cells

**Figure 1:**
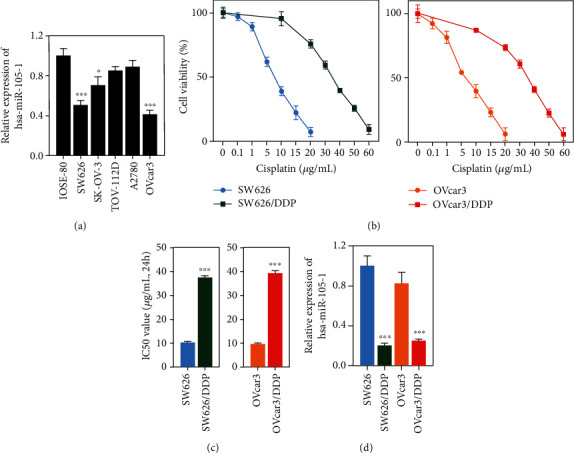
Hsa-miRNA-105-1 was abnormally expressed in cisplatin-resistant cell lines. (a) The levels of Hsa-miRNA-105-1 expression in a normal ovarian epithelial cell line (IOSE-80) and five OC cell lines (SW626, SK-OV-3, TOV-112D, A2780, and OVcar3). (b) The viability of cisplatin-sensitive and cisplatin-resistant cells when exposed to different cisplatin concentrations. (c) The IC_50_ values for wild type and cisplatin-resistant cells treated with cisplatin. (d) The levels of hsa-miRNA-105-1 expression in wild type and cisplatin-resistant cells. *n* = 3, ^∗^*P* < 0.05, ^∗∗∗^*P* < 0.01, and paired *t*-test.

qPCR assays were performed to analyze the levels of Hsa-miR-105-1 expression in normal cells and OC cells. The results showed that the levels of Hsa-miR-105-1 in 3 of the 5 OC cell lines were significantly lower than those in the IOSE-80 cell line (Figure 1(a)). SW626 and OVcar3 cells, which had the lowest levels of Hsa-miR-105-1, were chosen to establish cisplatin-resistant OC cell lines designated as SW626/DDP and OVcar3/DDP, respectively. The viability of the paternal cells and their resistance to different concentrations of cisplatin were detected by CCK-8 assays. The results showed that cisplatin-resistant cells were more viable and less sensitive to cisplatin than their paternal cells (Figures 1(b) and 1(c)). Furthermore, qPCR assays showed that Hsa-miR-105-1 was expressed at significantly lower levels in the cisplatin-resistant cells than in the wild type cells (Figure 1(d)).

### 3.2. ANXA9, the Target Gene of Hsa-miR-105-1, Was Upregulated in Cisplatin-Resistant OC Cells

**Figure 2:**
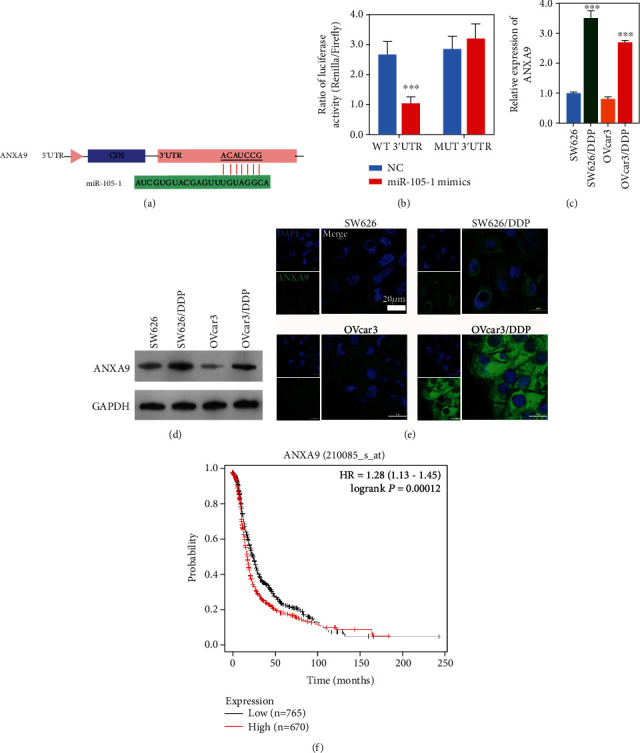
*ANXA9* was a target gene of Hsa-miRNA-105-1 in OC cells. (a) Schematic diagram showing the complementary sequences in the 3′ untranslated region (UTR) of ANXA9 mRNA and seed sequence of Hsa-miRNA-105-1. (b) Luciferase activity (firefly luciferase normalized to Renilla luciferase) in cells transfected with the wild-type or mutant ANXA9 3′-UTR reporter construct (*n* = 3, ^∗∗∗^*P* < 0.01, and paired *t*-test). (c) The levels of ANXA9 mRNA expression in parental and cisplatin-resistant OC cells (*n* = 3, ^∗∗∗^*P* < 0.01, and paired *t*-test). (d, e) The levels of ANXA9 protein expression in parental and cisplatin-resistant OC cells were detected by western blotting and immunofluorescence. Blue represents the cell nucleus stained with DAPI; green represents ANXA9 positive expression. (f) The effect of ANXA9 expression level on an OC patient's prognosis as indicated in the Kaplan-Meier Plotter database.

In order to investigate the functional mechanism of Hsa-miR-105-1, the TargetScan and Starbase databases were used to predict its putative target genes. Both analyses suggested *ANXA9* as a possible target gene for Hsa-miR-105-1 (Figure 2(a)). To confirm whether *ANXA9* was the real target of Hsa-miR-105-1, we generated a firefly luciferase reporter plasmid harboring either the wild-type or Hsa-miR-105-1 seed mutant 3′UTR of *ANXA9*. Luciferase reporter assays showed that the luciferase activity of the wild-type 3′UTR with Hsa-miR-105-1 was significantly lower than that of the control, confirming that *ANXA9* was the actual target gene of Hsa-miR-105-1 (Figure 2(b)). Furthermore, qRT-PCR, western blot, and immunofluorescence assays were used to verify the relationship between Hsa-miR-105-1 and *ANXA9*. Those results revealed that the levels of ANXA9 mRNA expression were significantly higher in the cisplatin-resistant cells when compared with the wild-type cells, which was opposite to the expression of Hsa-miR-105 (Figure 2(c)). Furthermore, the levels of ANXA9 protein expression were also significantly upregulated in the cisplatin-resistant cells (Figures 2(d) and 2(e)). Finally, data obtained from the Kaplan-Meier Plotter database (https://kmplot.com/analysis/index.php?p=service&cancer=ovar) revealed that a high level of *ANXA9* expression was always associated with a poor prognosis for OC patients (Figure 2(f)).

### 3.3. Downregulation of Hsa-miR-105 Increased ANXA9 Expression and Promoted OC Cell Cisplatin-Resistance

**Figure 3:**
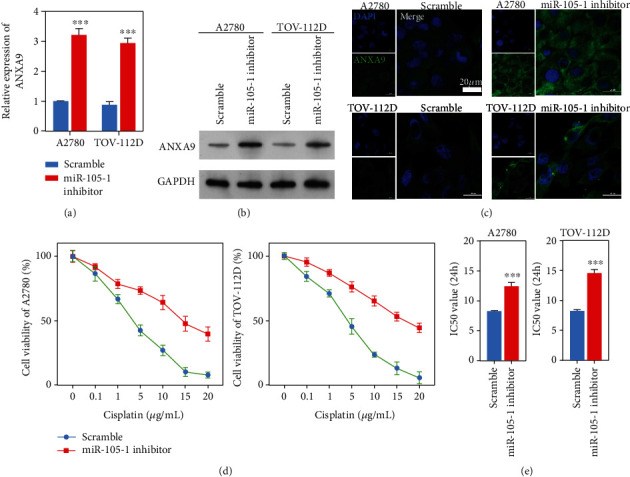
Downregulation of Hsa-miR-105-1 promoted *ANXA9* expression. A2780 and TOV-112D cells were transfected with scramble or the Hsa-miR-105-1 inhibitor. (a) The levels of ANXA9 mRNA expression as detected by the QPCR assay (*n* = 3, ^∗∗∗^*P* < 0.01, and paired *t*-test). (b) The levels of ANXA9 protein expression as detected by the western blotting. (c) The distribution of ANXA9 was shown by immunofluorescence. (d) The viability of scramble- or HAS-miR-105-1-treated A2780 or TOV-112D cells after exposure to different concentrations of cisplatin, as detected by the CCK8 assay. (e) The IC_50_ values for A2780 and TOV-112D cells treated with the scramble or Hsa-miR-105-1 inhibitor when exposed to cisplatin (*n* = 3, ^∗∗∗^*P* < 0.01, and paired *t*-test).

**Figure 4:**
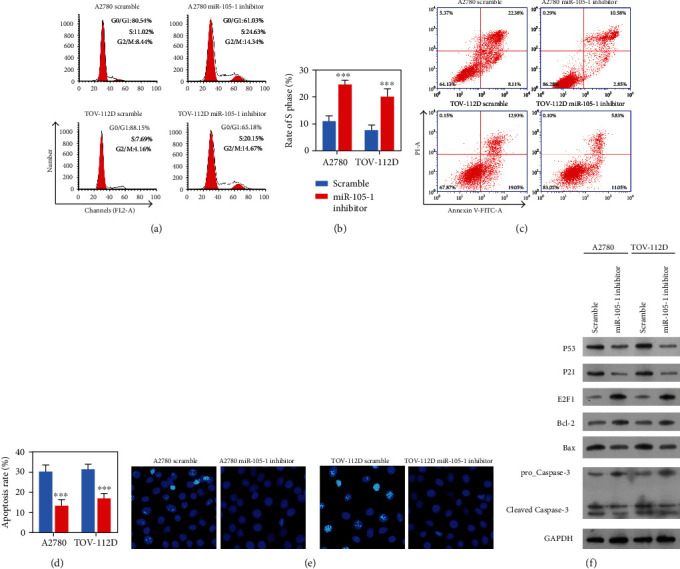
Downregulation of Hsa-miR-105-1 in OC cells induced resistance to cisplatin. A2780 and TOV-112D cells were transfected with the scramble or Hsa-miR-105-1 inhibitor and then stimulated with cisplatin (1 *μ*g/mL). (a, b) The cell cycle distributions of A2780 and TOV-112D cells as detected by flow cytometry. (c, d) The percentages of apoptotic A2780 and TOV-112D cells were determined by annexin V-FITC/PI staining and flow cytometry. (e) Cell apoptosis was detected by Hoechst staining. (f) The levels of P53, P21, E2F1, Bcl-2, Bax, and caspase-3 protein expression were examined by western blotting. ^∗∗∗^*P* < 0.01 and paired *t*-test.

To investigate the function of Hsa-miR-105-1 in OC cells, A2780 and TOV-112D cells, which had relatively high levels of Hsa-miR-105-1 expression, were transfected with a Hsa-miR-105-1 inhibitor. Subsequent qRT-PCR and western blot assays revealed that transfection with the Hsa-miR-105 inhibitor upregulated *ANXA9* expression at both mRNA and protein levels (Figures 3(a) and 3(b)). Immunofluorescence assays also demonstrated that the Hsa-miR-105-1 inhibitor could increase ANXA9 protein expression (Figure 3(c)). Meanwhile, cells transfected with the Hsa-miR-105-1 inhibitor exhibited a high resistance to cisplatin, as determined by CCK-8 assays (Figure 3(d)). The IC_50_ values of the miR-105-1 inhibitor-transfected cells were significantly higher than those of cells in scramble groups (Figure 3(e)). Next, we stimulated the cells with cisplatin (1 *μ*g/mL), and subsequent flow cytometry analyses showed that a lower percentage of cells treated with the Hsa-miR-105-1 inhibitor was in G0/G1 phase and a higher percentage was in S and G2/M phases when compared with cells treated with the scramble (Figures 4(a) and 4(b)). Furthermore, treatment with the Hsa-miR-105-1 inhibitor suppressed the cell apoptosis induced by cisplatin (Figures 4(c) and 4(d)). Hoechst staining showed that cells treated with the Hsa-miR-105-1 inhibitor had fewer bright fluorescent spots, which indicated less cell apoptosis (Figure 4(e)). In addition, western blot studies showed that downregulation of Hsa-miR-105-1 decreased the levels P53, P21, Bax, and Cleaved Caspase-3 protein expression and increased the levels of E2F1 and Bcl-2 protein expression (Figure 4(f)). These results indicated that the downregulation of Hsa-miR-105-1 could enhance cisplatin-resistance in OC cells.

### 3.4. Upregulation of Hsa-miR-105 Increased the Sensitivity of Cisplatin-Resistant Cells to Cisplatin

**Figure 5:**
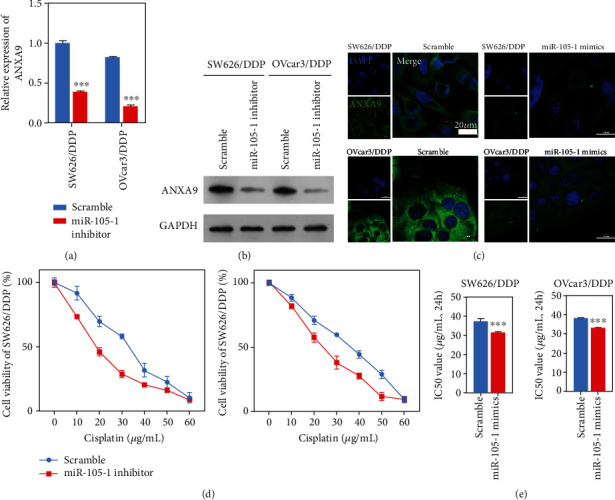
Upregulation of Hsa-miR-105-1 inhibited *ANXA9* expression in cisplatin-resistant OC cells. SW626/DDP and OVcar3/DDP cells were transfected with the scramble or Hsa-miR-105-1 mimics. (a) The levels of *ANXA9* expression as detected by qPCR assays (*n* = 3, ^∗∗∗^*P* < 0.01, and paired *t*-test). (b) The levels of ANXA9 protein expression as detected by western blotting. (c) The distribution of ANXA9 was shown by immunofluorescence. (d) The viability of scramble or Has-miR-105-1 mimic-treated SW626/DDP or OVcar3/DDP after exposure to different concentrations of cisplatin, as detected by the CCK8 assay. (e) The IC_50_ values for SW626/DDP and OVcar3/DDP cells treated with the scramble or Hsa-miR-105-1 mimics in cisplatin (*n* = 3, ^∗∗∗^*P* < 0.01, and paired *t*-test).

**Figure 6:**
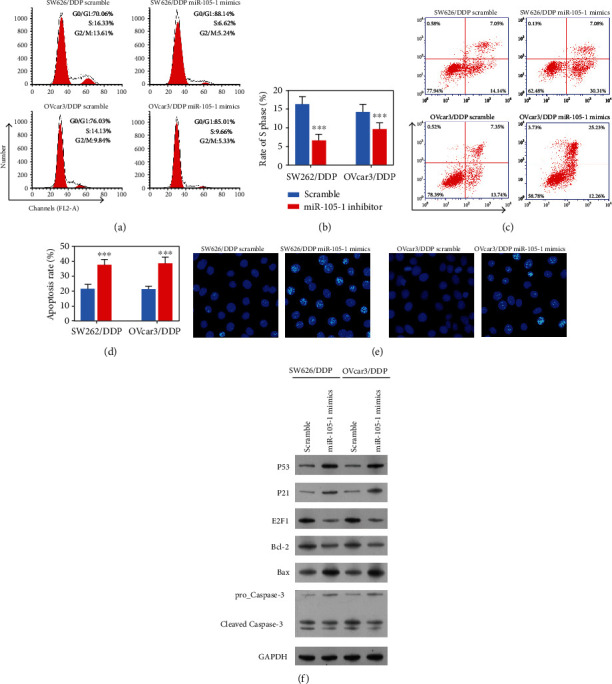
Upregulation of Hsa-miR-105-1 reduced the cisplatin resistance of SW626/DDP and OVcar3/DDP cells. SW626/DDP and OVcar3/DDP cells were transfected with the scramble or Hsa-miR-105-1 mimics and then stimulated with cisplatin (10 *μ*g/mL). (a, b) The cell cycle distributions of SW626/DDP and OVcar3/DDP cells were detected by flow cytometry. (c, d) The percentages of apoptotic SW626/DDP and OVcar3/DDP cells were determined by annexin V-FITC/PI staining and flow cytometry. (e) Cell apoptosis was detected by Hoechst staining. (f) The levels of P53, P21, E2F1, Bcl-2, Bax, and caspase-3 protein expression were examined by western blotting. ^∗∗∗^*P* < 0.01 and paired *t*-test.

To determine whether cisplatin resistance could be reversed by Hsa-miR-105-1, we upregulated the levels of Hsa-miR-105-1 in cisplatin-resistant OC cells, SW626/DDP cells, and OVcar3/DDP cells. Subsequent qRT-PCR analyses revealed that Hsa-miR-105-1 mimics inhibited *ANXA9* expression at the mRNA level, and western blot and immunofluorescence assays showed that Hsa-miR-105-1 mimics also inhibited the expression of ANXA9 protein (Figures 5(a)–5(c)). Transfection of OC cells with Hsa-miR-105-1 mimics decreased the viability of cisplatin-resistant cells treated with cisplatin (Figure 5(d)), as the IC_50_ values of the Hsa-miR-105-1 mimic-transfected cells were significantly lower than those of scramble group cells (Figure 5(e)). After simulation with 10 *μ*g/mL of cisplatin, flow cytometry analyses showed that a higher percentage of cells treated with Hsa-miR-105-1 mimics was in G0/G1 phase, and significantly lower percentages were in S and G2/M phases when compared to cells treated with the scramble (Figures 6(a) and 6(b)). Flow cytometry and Hoechst stain assays both showed that transfection with Hsa-miR-105-1 mimics promoted the cell apoptosis induced by cisplatin (Figures 6(c)–6(e)). In addition, western blot studies showed that upregulation of Hsa-miR-105-1 increased the levels of P53, P21s, and Bax expression and decreased the levels of E2F1 and Bcl-2 expression (Figure 6(f)). These results indicated that upregulation of Hsa-miR-105-1 could restore cisplatin sensitivity in cisplatin-resistant OC cells.

## 4. Discussion

The incidence and mortality rate of OC have increased in recent years. While cisplatin-based therapy is widely used to treat OC, the development of resistance to cisplatin has emerged as a major obstacle in treating ovarian cancers. Therefore, it is important to understand the mechanism responsible for cisplatin resistance if this major clinical problem is to be overcome. Here, we found that Hsa-miR-105-1 was expressed at abnormally low levels in cisplatin-resistant cells, and its target gene was *ANXA9*.

miRNAs not only function as tumor suppressors or oncogenes but also modulate chemoresistance in a variety of cancers. In recent years, it has become increasingly popular to study the roles played by various miRNAs in cancer chemoresistance. In OC, many miRNAs, such as miR-132 [10], miR-142 [11], miR-186 [12], and miR-195 [13] have been reported to modulate cisplatin-resistance. Studies conducted on miR-105 have mainly focused on its role as a prognostic marker and its ability to modulate cell proliferation, apoptosis, and metastasis [14–17]. To our knowledge, the relationship between miR-105 and cisplatin-resistance has only been explored in triple-negative breast cancer (TNBC), where it was shown to activate Wnt/*β*-catenin signaling by downregulating SFRP1 and thereby promote chemoresistance in TNBC [18]. In this study, we demonstrated that Hsa-miR-105-1 expression was suppressed in cisplatin-resistant OC cells, and an upregulation of Hsa-miR-105-1 could restore cisplatin sensitivity in OC cells.

miRNAs often perform their functions by binding to the 3′UTR of their downstream target mRNAs [17]. In our study, a bioinformatics analysis suggested that Hsa-miR-105-1 could directly bind to ANXN9 mRNA. Luciferase reporter assays demonstrated that overexpression of Hsa-miR-105-1 significantly repressed luciferase activity, indicating that ANXA9 mRNA possessed the Hsa-miR-105-1 binding site. In accordance with data from luciferase reporter assays, we found that treatment with an Hsa-miR-105-1 inhibitor significantly promoted *ANXA*9 expression, while treatment with Hsa-miR-105-1 mimics inhibited ANXA9 expression in OC cells.

ANXA9 is a member of the annexin family, which contains evolutionarily conserved proteins characterized by their ability to interact with membrane phospholipids in a Ca^2+^-dependent manner. This property most likely accounts for how annexin assists in regulating membrane organization and permeability and is thought to depend on so-called type II Ca^2+^ binding sites found in annexin proteins. In particular, numerous members of the annexin A subgroup are closely associated with cancer development [19–21]. However, ANXA9 has been studied far less than other annexins. *ANXA9*, initially called annexin 31, was first identified in the expressed sequence tags (EST) database [22]. The *ANXA9* gene (size, 8,233 bp) contains 14 exons, is located on human chromosome 1q21, and encodes a chain of 345 amino acids [23, 24]. The ANXA9 protein is a unique member of the annexin family because its intracellular activity does not appear to be regulated by calcium [24, 25]. Its closest evolutionary relatives are ANXA1 and ANXA2 [26, 27], and members of this clade are thought to function in organizing and regulating membrane/cytoskeleton linkages [27, 28].

Few studies have analyzed the expression of ANXA9 in cancers. Miyoshi et al. [29] reported that a high level of ANXA9 mRNA predicted a poor prognosis for patients with colorectal cancer (CRC). Another study suggested that ANXA9 might promote the invasion and metastasis of CRC by regulating genes associated with tumor invasion and metastasis [6]. In breast cancer, *ANXA9* gene expression is associated with bone metastasis and a patient's prognosis [7, 8]. In head and neck squamous cell carcinomas, the level of *ANXA9* is frequently altered and associated with the tumor's differentiation grade, suggesting that ANXA9 is associated with the pathogenesis of these cancers [9]. The above studies suggest that ANXA9 plays roles in tumor cell invasion and metastasis. In this study, we found that when *ANXA9* expression was upregulated by the Hsa-miR-105-1 inhibitor, cisplatin-resistance became enhanced, cell division was increased, and cell apoptosis was repressed. In contrast, when *ANXA9* was downregulated by Hsa-miR-105-1 mimics, cisplatin-resistance was restored, the OC cells remained in G0/G1 phase, and the rate of cell apoptosis increased. Notably, although our *in vitro* studies suggest a possible targeted therapy role for Hsa-miR-105-1 in treating cisplatin-resistant OC, *in vivo* studies of how Hsa-miR-105-1 functions in OC are still lacking. Subsequent *in vivo* studies that investigate the role played by Hsa-miR-105-1 in the cisplatin-resistance of OC may lead to the development of drugs that target the Hsa-miR-105-1/*ANXA9* axis.

## 5. Conclusion

In conclusion, the present study showed that Hsa-miR-105-1 mediates the cisplatin sensitivity of OC cells by targeting *ANXA9* and that Hsa-miR-105-1 expression is repressed in OC cells. Furthermore, a downregulation of Hsa-miR-105-1 expression enhanced cisplatin-resistance and decreased the apoptosis rate of OC cells, while an upregulation of Hsa-miR-105-1 expression could restore OC cell sensitivity to cisplatin. Our results provide new insights on how to overcome acquired resistance to cisplatin treatment and may serve to improve the therapeutic outcomes of patients with cisplatin-resistant OC.

## Data Availability

The datasets supporting the conclusions of this article are included within the article.
